# Retraction Note: Mining and urbanization affect river chemical water quality and macroinvertebrate communities in the upper Selenga River Basin, Mongolia

**DOI:** 10.1007/s10661-024-13042-x

**Published:** 2024-09-02

**Authors:** Dashdondog Narangarvuu, Tuuguu Enkhdul, Erdenesukh Erdenetsetseg, Enkhbat Enkhrii-Ujin, Khurtsbaatar Irmuunzaya, Gunsmaa Batbayar, Khurelpurev Oyundelger, Rita Sau-Wai Yam, Martin Pfeiffer

**Affiliations:** 1 Division of Ecology, National University of Mongolia, Ulaanbaatar, Mongolia; 2 Department of Environment and Forest Engineering, National University of Mongolia, Ulaanbaatar, Mongolia; 3 College of Environmental Studies, National Dong Hwa University, Shoufeng, Taiwan; 4 Department of Aquatic Ecosystem Analysis and Management (ASAM), Helmholtz Centre for Environmental Research-UFZ, Magdeburg, Germany; 5 Present Address: ADAPT Project, Ulaanbaatar, Mongolia; 6 Department of Botany, Senckenberg Museum of Natural History Görlitz, Görlitz, Germany; 7 Department of Bioenvironmental Systems Engineering, National Taiwan University, Taipei, Taiwan; 8 Department of Biogeography, University of Bayreuth, Universitätsstraße 30, 95447 Bayreuth, Germany

**Retraction Note: Environmental Monitoring and Assessment (2023) 195:1500**

10.1007/s10661-023-12022-x

The authors have retracted this article after they became aware that the units of measurement for five elements (B, Sr, Mo, U, V) were erroneously given as mg/l instead of µg/l in the measurements of chemical water quality. Consequently, the level of contamination by these elements was significantly overestimated. This error affects the results reported in Figure 3b and Table C1, as well as the Discussion section. The authors have been invited to submit a revised version of this article for peer review.

All authors agree with this retraction.

